# High Mobility Group Box1 Protein Is Involved in Endoplasmic Reticulum Stress Induced by* Clostridium difficile* Toxin A

**DOI:** 10.1155/2016/4130834

**Published:** 2016-08-07

**Authors:** Ji Liu, Yi Ma, Chun-Li Sun, Shan Li, Ju-Fang Wang

**Affiliations:** ^1^School of Bioscience & Bioengineering, South China University of Technology, Guangzhou 510006, China; ^2^Guangdong Province Key Laboratory of Fermentation and Enzyme Engineering, South China University of Technology, Guangzhou 510006, China

## Abstract

High Mobility Group Box1 (HMGB1), a damage-associated inflammatory factor, plays an important role in the pathogenesis of numerous chronic inflammatory and autoimmune diseases. In this study, the role of the HMGB1 in TcdA-induced ER stress was identified.* Clostridium difficile* toxin A is one of the major virulence factors of* C. difficile* infection (CDI) and has been proved to induce apoptotic cell death through ER stress. Our results showed that HMGB1 might play an important role in the TcdA-induced ER stress and unfolded protein response. HMGB1 activated molecular markers and induced the C/EBP homologous protein upregulation (CHOP). This study may provide the essential information for better understanding of the molecular mechanisms involved in CDI.

## 1. Introduction

*Clostridium difficile* is a Gram-positive, spore-forming anaerobic bacterium. It is responsible for primary and recurrent antibiotic-associated diarrhea and pseudomembranous colitis [1]. The recent emergence of hypervirulent strains has caused a rapid increase in the incidence of* C. difficile *infection (CDI) worldwide. Two exotoxins,* Clostridium difficile* toxin B (TcdB) and toxin A (TcdA), are key virulent factors of CDI [2, 3]. TcdA is highly cytotoxic, inducing the damage of intestinal epithelial cells and the release of inflammatory cytokines as well as trigging inflammatory and immune response [4–6]. Previous studies showed that TcdA could activate ERK2 and p38 MAP kinases in human monocytic cells and induce apoptotic cell death through ER stress [7].

High Mobility Group Box1 (HMGB1), the first identified member of the HMGB family, highly conserved in evolution, is described originally as a nuclear DNA-binding protein [8–10]. HMGB1 was identified as an important extracellular mediator of inflammation [11, 12]. Within the nucleus, HMGB1 maintains chromosomal structure and regulates DNA damage responses [13]. However, under a variety of stressful situations, HMGB1 is translocated to the cytosol, and is released into the extracellular coordinating inflammation, immunity, and other local cellular processes [14]. The recent discovery of extracellular HMGB1 as a proinflammatory mediator by TcdA-induced acute inflammation and intestinal damage already has been reported in our laboratory previously [15].

The endoplasmic reticulum (ER) that plays an essential role in multiple cellular processes encompasses about half the total membrane area and one-third of the newly translated proteins in a typical eukaryotic cell [16, 17], and it is an organelle that plays an essential role in multiple cellular processes. Live cells start a homeostatic signaling network named unfolded protein response (UPR), involving three stress transducer proteins, namely, protein kinase RNA-like ER kinase (PERK), inositol-requiring protein 1*α* (IRE1*α*), and activating transcription factor 6 (ATF6), each of which activates its own unique cascade of downstream events to regulate metabolism and survival of cells [18].

The apoptotic properties of TcdA have been confirmed in different kinds of cell lines [5, 6]. And TcdA-induced ER stress reactions also have been clearly described in cells [19, 20]. In the paper, the potential role of HMGB1 involved in TcdA-induced ER stress was identified which could reveal its underlying mechanism making a contribution to the development of CDI therapies.

## 2. Materials and Methods

### 2.1. Cell Culture and TcdA Purification

CT26 (the murine colon adenocarcinoma cell line) was obtained from American Type Culture Collection (Manassas, USA) and cultured in Dulbecco's Modified Eagle's medium (GIBCO, Carlsbad, USA) containing 10% fetal bovine serum (GIBCO), 100 U/mL penicillin, 100 *μ*g/mL streptomycin, 2 mM L-glutamine, and 1 mM sodium pyruvate (GIBCO).

The full-length wild-type recombinant TcdA plasmid was kindly provided by Dr. Feng (University of Maryland at Baltimore, Baltimore, USA). TcdA was expressed and purified according to the protocol reported by Sullivan et al. [21]. The highly purified recombinant TcdA appeared as a single band on sodium dodecyl sulfate polyacrylamide gels (data not shown). rHMGB1 was purchased from Uscn Life Science Inc. (Wuhan, China).

### 2.2. Cell Rounding Assay

CT26 cells were seeded in 96-well plates (1 × 10^4^ cells/well) and then were treated with TcdA (10 ng/mL) for 4 h or pretreated with 100 *μ*M glycyrrhizin (30 min) before TcdA exposure. Cell rounding was visualized by phase-contrast microscopy. Each concentration was tested in triplicate for overall cell rounding, and the experiments were repeated three times.

### 2.3. Western Blot Analysis

Total protein extracts were prepared and separated on 12% SDS-PAGE and electrophoretically transferred to nitrocellulose filter membranes. The membranes were blocked with 5% bovine serum albumin. And then the membranes were incubated overnight at 4°C with the indicated primary antibodies. The dilutions of antibodies were prepared as follows: HMGB1 (CST number 3935, 1 : 1000), BSA (Santa Cruz sc-50528, 1 : 1000), PERK (CST number 3192, 1 : 1000), CHOP (CST number 2895, 1 : 1000), IRE1*α* (CST number 3294, 1 : 1000), ATF6 (Abcam ab62576, 1 : 2000), Bcl_2_ (Santa Cruse number KO112, 1 : 500), and *β*-actin (CST number 5125, 1 : 1000). After washing, membranes were treated with horseradish peroxidase-conjugated secondary antibodies (1 : 2000; CST, number 7074, USA) and then the bands were visualized using the enhanced chemiluminescence kit (Thermo, number 34080, USA).

### 2.4. Statistical Analysis

Unless indicated, experiments were repeated at least three times. Data were expressed as the mean ± SEM. Data were analyzed using Prism v.5.03 (GraphPad Software, San Diego, USA). Statistical significance was assessed by one-way analysis of variance (ANOVA) followed by Turkey's test or two-way repeated-measures ANOVA. *P* < 0.05 was considered as statistically significant. 

## 3. Results

### 3.1. TcdA Exposure Induces HMGB1 Release from CT26 Cells

The effect of TcdA on CT26 cells was examined by cell rounding assay as evidenced by morphological changes and survival inhibition of cells.

**Figure 1:**
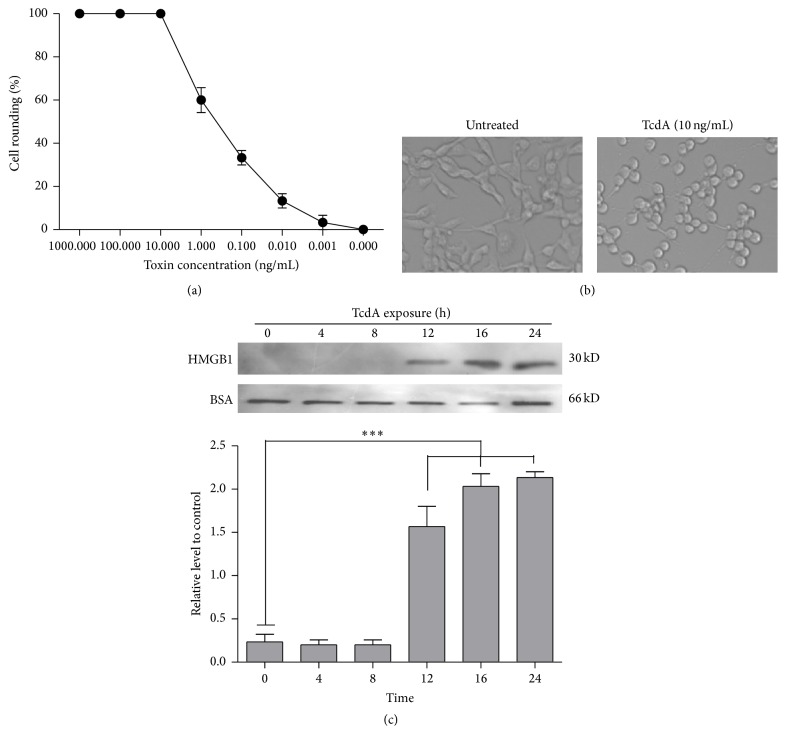
TcdA induces the release of HMGB1 from CT26 cells. (a) CT26 cells were treated with different concentrations of TcdA for 4 h, and the rate of cell rounding was calculated. (b) CT26 cells were exposed to the medium (cell control) or the TcdA for 4 h. The percentage of cells affected (cell rounding) was observed under a phase-contrast microscope. (c) CT26 cells were exposed to 10 ng/mL TcdA for the indicated time intervals, and HMGB1 levels in the culture medium were detected by western blot analysis using BSA as a loading control. ^*∗∗∗*^ *P* < 0.001.

CT26 cells treated with TcdA were examined, which showed that TcdA induced cell rounding in a dose dependent manner, with 60% cell rounding observed after exposure to 1 ng/mL TcdA and 100% cell rounding after exposure to 10 ng/mL for 4 h (Figure 1(a)). And the results showed that, after exposure to 10 ng/mL TcdA, the morphology of CT26 cells changed from fusiform (control) to rounding (Figure 1(b)).

To measure HMGB1 secretion in response to TcdA, CT26 cells were cultured in the presence of 10 ng/mL TcdA and the medium was collected at the indicated times. Western blot analysis showed that the release of HMGB1 induced by TcdA in medium was increased in a time-dependent manner after 12 h of exposure (Figure 1(c)).

### 3.2. Exogenous rHMGB1 Induces ER Stress

**Figure 2:**
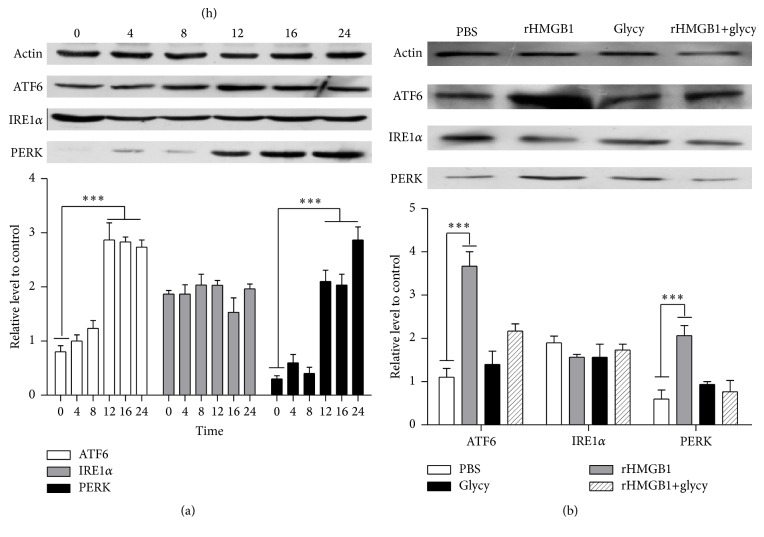
Glycyrrhizin prevents HMGB1-induced ER stress. (a) Protein expressions of IRE1*α*, ATF6, and PERK in CT26 cells treated with HMGB1 for the indicated time were measured by western blot. (b) rHMGB1 combined with glycyrrhizin was incubated with CT26 cells for 12 h and the protein expressions of IRE1*α*, ATF6, and PERK were detected by western blot analysis. Actin was used as the loading control. Data represent the mean of three independent experiments. ^*∗∗∗*^ *P* < 0.001.

To determine whether HMGB1 is involved in ER stress, rHMGB1 was used to verify the assumption. CT26 cells were incubated with 1 ng/mL rHMGB1 and were collected at different time points (0, 4, 8, 12, 16, and 24 h). IRE1, ATF6, and PERK branches were detected using western blot. As shown in Figure 2(a), the expressions of the ATF6 and PERK in cells were markedly elevated in a time-dependent manner at 12 h of rHMGB1 exposure, in contrast to those of the PBS group, and the expression levels of PERK were detected after 4 h and continued increasing until the end of the experiment; the content of ATF6 was enhanced to the maximum value at 12 h and did not recover at 24 h, whereas the protein expression of IRE1*α* had no change.

Furthermore, to investigate the involvement of HMGB1 in ER stress, glycyrrhizin, the HMGB1 inhibitor [22], was added to the CT26 cells to inhibit activity of rHMGB1. After exposure to rHMGB1 and glycyrrhizin for 12 h, cells were collected and proteins were extracted to evaluate the expression level of IRE1*α*, ATF6, and PERK by western blot. As shown in Figure 2(b), as expected, the ATF6 and PERK proteins expressions were decreased in mixture group (the mixture of 20 *μ*M glycyrrhizin and rHMGB1), in contrast to those of the rHMGB1 group. No significant difference was observed between the glycyrrhizin group and the mixture group, which was similar to that of PBS group, whereas the IRE1*α* protein had not significantly changed in different groups.

And the statistical analysis showed that the level of the ATF6 and PERK proteins in rHMGB1 treatment group had significantly increased in contrast to those of PBS (*P* < 0.0001). In contrast, there was no significant difference in the expression of IRE1*α*.

### 3.3. Glycyrrhizin Alleviates TcdA-Induced Cell Damage

**Figure 3:**
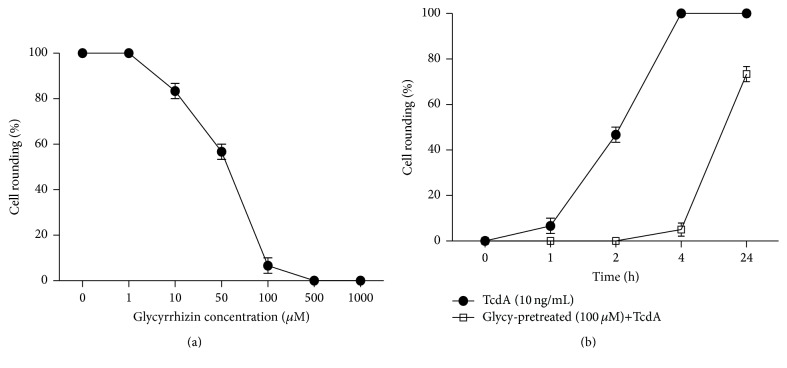
Glycyrrhizin prevents TcdA-induced cell rounding. CT26 cells were treated with glycyrrhizin and TcdA, and cell rounding rate was measured. (a) CT26 cells were pretreated with different concentrations of glycyrrhizin, followed by 10 ng/mL TcdA for 4 h, and cell rounding was quantified. (b) CT26 cells were treated with glycyrrhizin 30 min before TcdA exposure, and the rate of cell rounding was determined.

HMGB1 is released from cells exposed to TcdA and further induces cell damage. So we pretreated the cells with glycyrrhizin, the HMGB1 inhibitor [22], prior to TcdA exposure to inhibit the activity of the subsequently secreted HMGB1 and further observed the relationship between HMGB1 and TcdA-induced cell damage. As shown in Figure 3(a), CT26 cells were treated with different concentration of glycyrrhizin, prior to 10 ng/mL TcdA exposure; 100 *μ*M glycyrrhizin could completely inhibit the toxicity of TcdA, whereas glycyrrhizin alone did not induce cell rounding and the cells showed normal morphology. CT26 cells were exposed to 10 ng/mL TcdA for different times; 50% cell rounding appeared after exposure for 2 h and 100% cell rounding appeared after exposure for 4 h, whereas there was no cell rounding after 4 h and only 50% cell rounding occurred at 24 h when cells were pretreated with 100 *μ*M glycyrrhizin before exposure to TcdA (Figure 3(b)).

### 3.4. Glycyrrhizin Pretreatment Affects TcdA-Induced ER Stress

Further experiments were carried out to investigate if HMGB1 was involved in endoplasmic reticulum stress induced by* Clostridium difficile* toxin A. Treating the cells with glycyrrhizin prior to TcdA exposure which inhibited the activity of the subsequently secreted HMGB1. CT26 cells were incubated with glycyrrhizin (100 *μ*M) for 30 minutes before TcdA exposure and extracted proteins to determine the level of IRE1*α*, ATF6, and PERK by western blot.

**Figure 4:**
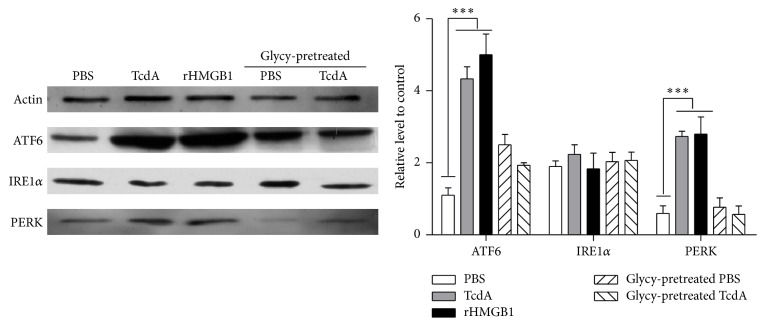
HMGB1 involves TcdA-induced ER stress. CT26 cells were pretreated with glycyrrhizin 30 min before TcdA exposure. And then CT26 cells were treated by TcdA for 12 h and the protein expressions of IRE1*α*, ATF6 and PERK were detected by western bolt analysis. Actin was used as the loading control. Data represent the mean of three independent experiments. ^*∗∗∗*^ *P* < 0.001.

As shown in Figure 4, ER stress markers were expressed after treatment of 10 ng/mL TcdA. The expression level of ATF6 and PERK proteins significantly increased, compared with pretreated glycyrrhizin group. The same result was observed in rHMGB1 group. By contrast, without TcdA exposure, the glycyrrhizin or PBS group's proteins expression significantly was decreased to normal levels, whereas the IRE1*α* protein was not significantly changed when the CT26 was treated with TcdA or pretreated glycyrrhizin.

The statistical analysis showed that there is notable difference between the expression levels of the ATF6 and PERK proteins in TcdA treatment group and those of the glycyrrhizin pretreatment group (*P* < 0.0001).

### 3.5. HMGB1 Involved Apoptotic ER Stress

In order to investigate the involvement of HMGB1 in TcdA-induced apoptotic ER stress, two experiments were designed and two key mediators of apoptosis ER stress marker, CHOP and Bcl_2_, were detected.

**Figure 5:**
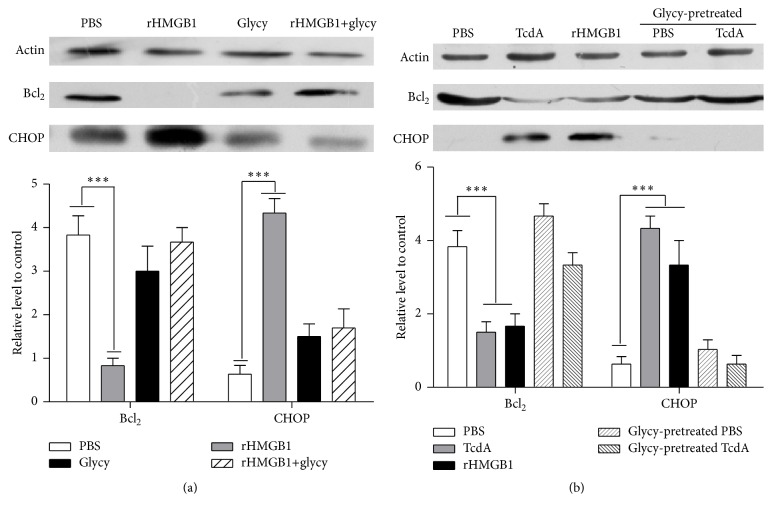
Glycyrrhizin reduces HMGB1-induced apoptotic ER stress. CT26 cells were pretreated with glycyrrhizin 30 min before HMGB1 exposure. And then CT26 cells were treated by HMGB1 for 12 h and collected for analysis. The proteins levels change of CHOP and Bcl_2_ were examined by western blotting. (a) Glycyrrhizin prevented HMGB1-induced apoptotic ER stress. (b) Glycyrrhizin reduced TcdA-induced apoptotic ER stress. Actin was used as the loading control. Data represent the mean of three independent experiments. ^*∗∗∗*^ *P* < 0.001.

rHMGB1 was used to verify the involvement of rHMGB1 in apoptotic ER stress. As shown in Figure 5(a), after exposure to rHMGB1 or the mixture of rHMGB1+glycyrrhizin for 12 h, proteins were extracted from the collected cells to evaluate the expression level of CHOP and Bcl_2_. CHOP protein expression was obvious upregulation which was treated with rHMGB1. In contrast, CHOP proteins expression significantly decreased in the mixture group. Bcl_2_ protein had significantly decreased in rHMGB1 group, compared with PBS group, and the Bcl_2_ expressions in the mixture group were not significantly different from those in the PBS group.

Further experiments were carried out to investigate if HMGB1 involved apoptotic ER stress by TcdA. CT26 cells were incubated with glycyrrhizin (100 *μ*M) for 30 minutes before TcdA exposure and extracted proteins to determine the level of CHOP and Bcl_2_. As expected, the CHOP, a key mediator of apoptotic ER stress, was significantly increased after treatment with 10 ng/mL TcdA (Figure 5(b)), compared with glycyrrhizin pretreatment group. The expression of Bcl_2_ in glycyrrhizin pretreatment group was apparently reduced, compared with that of the control. By contrast, without TcdA exposure, no significant difference of the CHOP and Bcl_2_ expression was observed between the glycyrrhizin and PBS group. Our results revealed that HMGB1 could be involved in apoptotic ER stress induced by TcdA.

## 4. Discussion

*Clostridium difficile* toxin A is one of the major virulence factors of* C. difficile *infection (CDI) and has been proved to induce apoptotic cell death through ER stress pathway by previous work [19, 20]. HMGB1 is stabilized nucleosomal structure and facilitates gene transcription in intracellular [8, 10]. Moreover, HMGB1 is massively released extracellularly and plays a cytokine-like function [12, 13]. Early studies have shown that HMGB1 is released from intestinal cell and involved in toxin-induced inflammation [15].

The results of our study demonstrated that HMGB1 is involved in endoplasmic reticulum stress induced by* Clostridium difficile* toxin A. And it proved that HMGB1 was released from the nucleus to culture medium after exposure of TcdA for 12 h. Glycyrrhizin can attenuate the activity of HMGB1. The results showed that glycyrrhizin pretreatment delayed the onset of TcdA-induced cell rounding (Figure 3(b)). These results imply that HMGB1 is probably involved in the cytotoxic and cytopathic effects of TcdA. Glycyrrhizin could modulate the ATF6 and PERK signaling pathways, leading to upregulation of the ATF6, PERK, and CHOP expressions and suppressing endoplasmic reticulum stress. The inhibitory effect of glycyrrhizin on ER stress is another possible mechanism by which glycyrrhizin prevents HMGB1-related cell damage and ER stress. Overall, it is supposed that late stage of release of HMGB1 or activity of HMGB1 has potential effects on ER stress.

TcdA could induce apoptosis of cells including intestinal epithelial cells [4, 5]. To investigate the interaction of HMGB1 and TcdA-induced apoptotic ER stress, we successfully demonstrated that rHMGB1-induced ER stress could be able to increase the level of CHOP and decrease the Bcl_2_ protein expression, which is similar to the effect of toxin A (Figure 5(a)), suggesting that the CHOP and Bcl_2_ proteins are involved in cell death induced by TcdA or rHMGB1. CHOP and Bcl_2_ are the key mediators involved in ER stress induced apoptosis. CHOP mediates cell death primarily through two mechanisms, alteration of the transcription of genes involved in apoptosis and oxidative stress [23, 24].

In summary, our data suggest that HMGB1 plays a role in endoplasmic reticulum stress induced by* Clostridium difficile* toxin A. It hints that HMGB1 can possibly be a potential candidate for therapies of CDI, which might represent a new approach in the development of new drugs for CDI.

## References

[1] Kyne L. (2010). Clostridium difficile—beyond antibiotics. *The New England Journal of Medicine*.

[2] Elliott B., Chang B. J., Golledge C. L., Riley T. V. (2007). *Clostridium difficile*-associated diarrhoea. *Journal of International Medicine*.

[3] Kelly C. P., Pothoulakis C., Lamont J. T. (1994). *Clostridium difficile* colitis. *The New England Journal of Medicine*.

[4] Yang G., Zhou B., Wang J. (2008). Expression of recombinant *Clostridium difficile* toxin A and B in *Bacillus megaterium*. *BMC Microbiology*.

[5] Matarrese P., Falzano L., Fabbri A. (2007). *Clostridium difficile* toxin B causes apoptosis in epithelial cells by thrilling mitochondria: involvement of ATP-sensitive mitochondrial potassium channels. *The Journal of Biological Chemistry*.

[6] Fiorentini C., Fabbri A., Falzano L. (1998). *Clostridium difficile* toxin B induces apoptosis in intestinal cultured cells. *Infection and Immunity*.

[7] Warny M., Keates A. C., Keates S. (2000). p38 MAP kinase activation by *Clostridium difficile* toxin A mediates monocyte necrosis, IL-8 production, and enteritis. *The Journal of Clinical Investigation*.

[8] Andersson U., Tracey K. J. (2011). HMGB1 is a therapeutic target for sterile inflammation and infection. *Annual Review of Immunology*.

[9] Yanai H., Ban T., Taniguchi T. (2011). Essential role of high-mobility group box proteins in nucleic acid-mediated innate immune responses. *Journal of Internal Medicine*.

[10] Štros M. (2010). HMGB proteins: interactions with DNA and chromatin. *Biochimica et Biophysica Acta—Gene Regulatory Mechanisms*.

[11] Wang H., Bloom O., Zhang M. (1999). HMG-1 as a late mediator of endotoxin lethality in mice. *Science*.

[12] Caffidi S. P., Misteli T., Bianchi M. E. (2002). Release ofvchromatin protein HMGB1 by necrotic cells triggers inflammation. *Nature*.

[13] Lange S. S., Mitchell D. L., Vasquez K. M. (2008). High mobility group protein B1 enhances DNA repair and chromatin modification after DNA damage. *Proceedings of the National Academy of Sciences of the United States of America*.

[14] Lotze M. T., Tracey K. J. (2005). High-mobility group box 1 protein (HMGB1): nuclear weapon in the immune arsenal. *Nature Reviews Immunology*.

[15] Liu J., Zhang B. L., Sun C. L., Li S., Wang J. F. (2016). High mobility group box1 protein is involved in acute inflammation induced by *Clostridium difficile* toxin A. *Acta Biochimica et Biophysica Sinica*.

[16] Alberts B., Johnson A., Lewis J., Raff M., Roberts K., Walter P. (2002). *Molecular Biology of the Cell*.

[17] Kaufman R. J. (1999). Stress signaling from the lumen of the endoplasmic reticulum: coordination of gene transcriptional and translational controls. *Genes and Development*.

[18] Schröder M., Kaufman R. J. (2005). ER stress and the unfolded protein response. *Mutation Research*.

[19] Chen X., Kokkotou E. G., Mustafa N. (2006). Saccharomyces boulardii inhibits ERK1/2 mitogen-activated protein kinase activation both in vitro and in vivo and protects against *Clostridium difficile* toxin A-induced enteritis. *The Journal of Biological Chemistry*.

[20] Qiu B., Pothoulakis C., Castagliuolo I., Nikulasson S., LaMont J. T. (1999). Participation of reactive oxygen metabolites in *Clostridium difficile* toxin A-induced enteritis in rats. *American Journal of Physiology—Gastrointestinal and Liver Physiology*.

[21] Sullivan N. M., Pellett S., Wilkins T. D. (1982). Purification and characterization of toxins A and B of *Clostridium difficile*. *Infection and Immunity*.

[22] Bromati C. R., Lellis-Santos C., Yamanaka T. S. (2011). UPR induces transient burst of apoptosis in islets of early lactating rats through reduced AKT phosphorylation via ATF4/CHOP stimulation of TRB3 expression. *American Journal of Physiology—Regulatory Integrative and Comparative Physiology*.

[23] Mollica L., De Marchis F., Spitaleri A. (2007). Glycyrrhizin binds to high-mobility group box 1 protein and inhibits its cytokine activities. *Chemistry & Biology*.

[24] Zhao L., Ackerman S. L. (2006). Endoplasmic reticulum stress in health and disease. *Current Opinion in Cell Biology*.

